# Repurposing Duloxetine as a Potent Butyrylcholinesterase Inhibitor: Potential Cholinergic Enhancing Benefits for Elderly Individuals with Depression and Cognitive Impairment

**DOI:** 10.1021/acsomega.4c05089

**Published:** 2024-08-21

**Authors:** Taher Darreh-Shori, Anurag T. K. Baidya, Medea Brouwer, Amit Kumar, Rajnish Kumar

**Affiliations:** † Division of Clinical Geriatrics, Centre for Alzheimer Research, Department of Neurobiology, Care Sciences and Society, Karolinska Institutet, NEO, Seventh Floor, 141 52 Stockholm, Sweden; ‡ Department of Pharmaceutical Engineering & Technology, Indian Institute of Technology (B.H.U.), Varanasi 221005, Uttar Pradesh, India

## Abstract

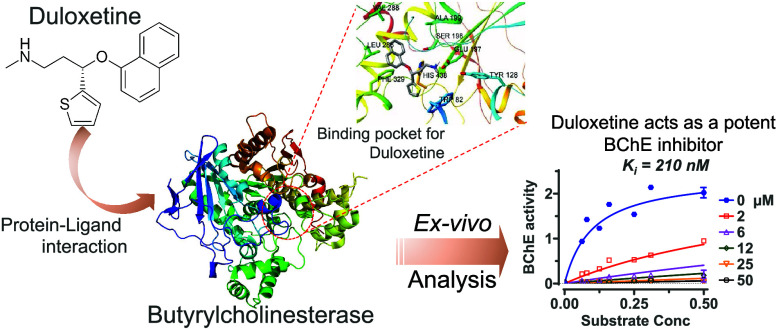

Despite the advent of new treatment strategies, cholinesterase inhibitors (ChEIs) are still the go-to treatment for dementia disorders. ChEIs act by inhibiting the main acetylcholine-degrading enzyme, acetylcholinesterase (AChE). Nonetheless, accumulating evidence indicates that the impact of inhibition of the sister enzyme, butyrylcholinesterase (BChE), could be even broader in older adults due to the multifaceted role of BChE in several biological functional pathways. Therefore, we employed an *in silico* modeling-based drug repurposing strategy to identify novel potent BChE inhibitors from the FDA drug database. This was followed by *in vitro* screening and *ex vivo* enzyme kinetic validation using human plasma samples as the source of BChE. The analysis revealed that the antidepressant drug, duloxetine, inhibited BChE with high selectivity in comparison to AChE. In contrast, two other antidepressants, namely, citalopram and escitalopram exhibited a weak to moderate activity. *Ex vivo* enzyme inhibition kinetic analyses indicated that duloxetine acted as a competitive inhibitor of BChE with an inhibition constant (*K*_i_) of 210 nM. This *K*_i_ value is comparable with 100–400 nM concentration of duloxetine following normal dosages in humans, thereby indicating that duloxetine should be able to induce a pharmacologically and biologically relevant *in vivo* inhibition of BChE. Additionally, we performed the enzyme inhibition kinetic assessment in parallel for ethopropazine, a known potent selective BChE inhibitor, and physostigmine, a dual inhibitor of AChE and BChE. These analyses indicated that duloxetine should be considered a potent BChE inhibitor since its *K*_i_ was comparable with ethopropazine (*K*_i_ = 150 nM) but was 4 times smaller than that of physostigmine (*K*_i_*=* 840 nM). In conclusion, this study reports the discovery of duloxetine being a highly potent selective competitive BChE inhibitor. This, in turn, indicates that duloxetine could be the choice of antidepressive treatment in older adults with both depressive and dementia symptoms since it may offer additional clinically beneficial effects *via* this secondary mode of cholinergic enhancing action.

## Introduction

Accumulating reports indicate that the acetylcholine-degrading enzyme, butyrylcholinesterase (BChE), could be an important target enzyme for improving various age- and disease-related deficits or dysfunctions in the central and peripheral cholinergic signaling system, such as the regulation of the immune system and/or the brain cognitive function in older adults and in patients with impaired cognition.^1−5^ Indeed, the reports indicate that BChE participates in the regulation of cholinergic signaling in the same manner as the main acetylcholine-degrading enzyme, i.e., acetylcholinesterase (AChE) is involved.^4^ Genetic studies on BCHE gene variants imply that carriers of the BCHE-K mutation show a delay in developing cognitive impairment, plausibly due to the fact that the BChE-K variant enzyme intrinsically exhibits about one-third the activity of the wild-type enzyme. In other words, subjects with the K variant of BChE show resistance against developing cognitive impairment through their 30% intrinsic BChE inhibition.^6^ Animal study on BChE inhibitors supports the notion that the selective inhibition of BChE may improve both the cognitive function as well as modulate the brain levels of β-amyloid peptides.^7^

Evidently, BChE can effectively hydrolyze acetylcholine and contribute greatly to normal cholinergic transmission than previously speculated.^8,9^ In the healthy human brain, AChE is the primary enzyme for the breakdown of acetylcholine, and the BChE activity accounts for only about 10% of the total cholinesterase activity.^10^ Nonetheless, there is a gradual decline in the AChE activity up to 45%, while the BChE activity increases by up to 40–90% with the progression of Alzheimer’s disease (AD).^9,11^ In addition, the BChE activity rather than the AChE activity correlates with the levels of astroglia biomarkers and proinflammatory cytokines in the cerebral spinal fluid (CSF) of patients with mild cognitive impairment (MCI) and mild AD.^1,6^ Altogether, the accumulated evidence emphasizes the importance of BChE as a suitable antidementia drug target both at the advanced age as well as the initial and late stages of dementia disorders.^1−6,8,9,11^

Another morbidity that is common among older adults is depression, which is manifested in terms of physical disabilities and cognitive deficit, resulting in a significant burden on the patients and their families.^12,13^ Antidepressants of choice are selective serotonin reuptake inhibitors (SSRIs) and serotonin-norepinephrine reuptake inhibitors (SNRIs).^14^ The racemate drug, citalopram, and its *S*-enantiomer, escitalopram, are two of the common SSRIs used as antidepressants in older adults. Duloxetine is a SNRI that has been tested in elderly populations with good results.^13,15^

The current indications for duloxetine use are the treatment of major depressive disorders, generalized anxiety disorders, neuropathic pain, and pain in association with diabetic peripheral neuropathy, musculoskeletal pain, and fibromyalgia. In some countries, it is also used for treatment of stress incontinence and urinary incontinence in women.^16^ Radioligand binding studies suggest that duloxetine inhibits both norepinephrine and 5-HT uptake carriers with *K*_i_ values of 2.1 and 0.53 nM, respectively. These studies also indicate that duloxetine lacks the pharmacologically relevant affinity for muscarinic, histaminergic, α1-adrenergic, serotonergic, dopaminergic, and opioid receptors.^17^ Its primary mechanism of action is therefore believed to be mediated by increasing the level of serotonin and/or norepinephrine in the brain.^16^ Pharmacokinetic studies indicate that duloxetine has a plasma *C*_max_ of 70–180 nM after a single dose of 30–90 mg. Following multiple doses of 30 mg twice daily, the maximum and minimum steady-state plasma concentrations of duloxetine are 142 and 84 nM, respectively.^18^ Similar studies show that the concentration of citalopram ranges between 86 and 857 nM in the plasma and between 40 and 295 nM in the CSF, depending on the daily dosage.^19^ The concentrations can be slightly higher in elderly populations than in young people.^20^ The mean plasma concentration of escitalopram is fairly like that of citalopram and varies depending on the dosage (194 and 608 nM, following repeated doses of 10 and 30 mg/day, respectively^21^).

In a study that primarily aimed at identifying new ligands of the key cholinergic enzyme, choline acetyltransferase (ChAT), we performed *in silico* analysis of a database of U.S. Food and Drug Administration (FDA)-approved drugs.^22,23^ Nonetheless, the *in silico* analysis revealed that some of the screened FDA drugs may have considerable activity on AChE and BChE enzymes.

In the current study, we report a detailed enzyme kinetic study together with *in silico* docking analyses on three of the identified drugs, namely, duloxetine, citalopram, and escitalopram. We show that duloxetine acts as a highly selective potent reversible BChE inhibitor, while citalopram and escitalopram exhibit weak to moderate potencies as BChE and/or AChE inhibitors. The accompanied *in silico* docking analyses reveal the fingerprints of the molecular interaction between the drugs and the amino acid residues at the binding sites on the enzymes. Given that evidence suggests that the selective inhibition of BChE may have a beneficial effect in dementia disorders^4^ and that depression and musculoskeletal pain as well as stress incontinence are relatively common in the elderly suffering from cognitive impairments, the treatment of these patients with duloxetine may be more beneficial than the treatment with other antidepressant drugs.

## Results

### *In Vitro* Screening of the Compounds

Duloxetine, citalopram, and escitalopram were tested in an *in vitro* assay at a single concentration for their inhibition of human AChE, human plasma BChE, and human recombinant ChAT enzymes. All of the drugs were tested at a final concentration of 100 μM.

**Figure 1:**
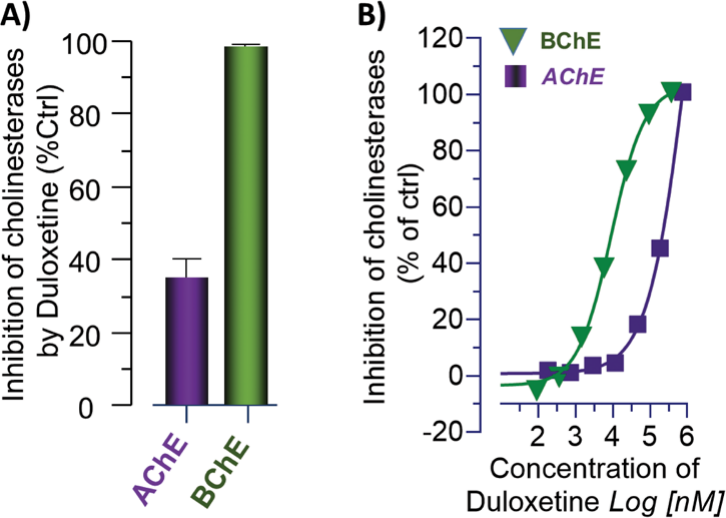
*In vitro* characterization of duloxetine following the *in silico* analyses against human cholinesterases. (A) Single 100 μM concentration screening of duloxetine against human acetylcholinesterase (AChE) and butyrylcholinesterase (BChE). (B) Half-maximal inhibitory concentration (IC_50_) analyses for duloxetine toward human AChE and BChE. The analyses were done at 0.5 and 5 mM concentrations of acetylthiocholine and butyrylthiocholine as preferred substrates of AChE and BChE, respectively. All data are shown as the percent of the enzymes’ activity in the presence of the solvent (vehicle control). Data are shown as mean ± standard deviation (SD). The IC_50_ analyses were done with GraphPad Prism 9.

This *in vitro* screening indicated that at 100 μM concentration, duloxetine fully inhibited the activity of plasma BChE, while AChE was inhibited by about 35% (Figure 1A). Next, we assessed the IC_50_ of duloxetine for plasma BChE at a substrate concentration of 5 mM. This resulted in an IC_50_ of 9.7 μM (with 7.6–12.5 μM, 95% CI, Figure 1B). The corresponding IC_50_ of duloxetine against human AChE was 112 μM (159–791 μM, 95% CI, Figure 1B) at a substrate concentration of 0.5 mM.

**Figure 2:**
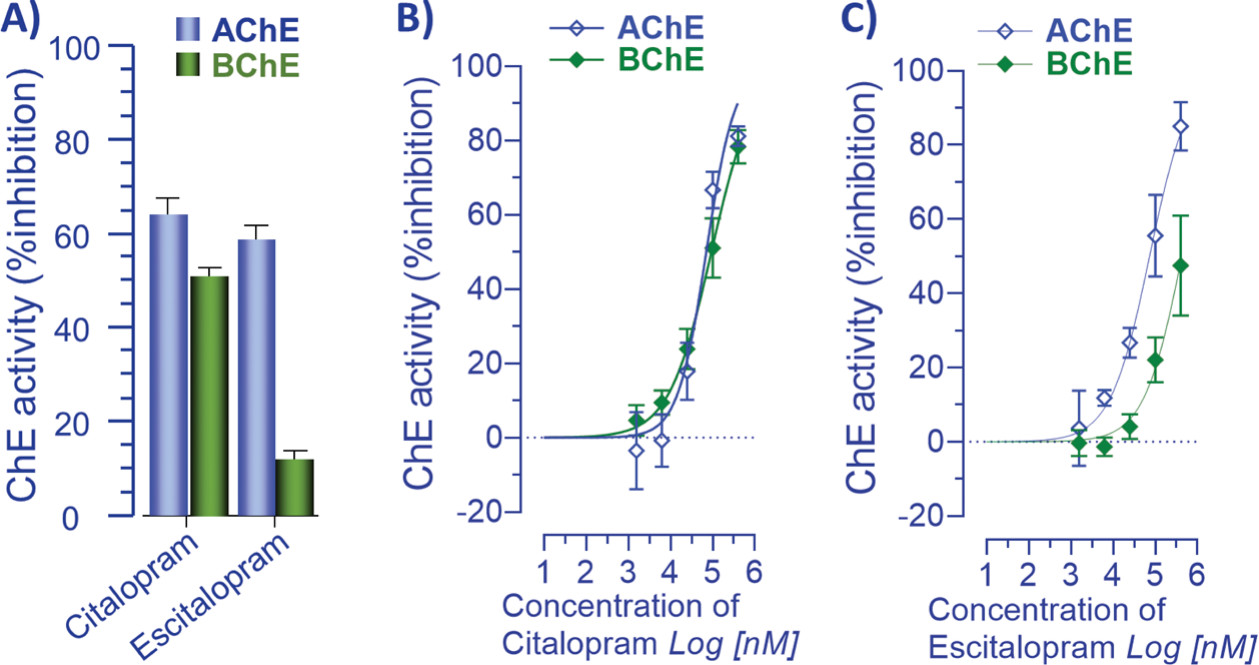
*In vitro* characterization of citalopram and escitalopram following the *in silico* analyses against human cholinesterases and choline acetyltransferase. (A) Single 100 μM concentration screening for citalopram and escitalopram against human acetylcholinesterase (AChE) and butyrylcholinesterase (BChE). (B) Half-maximal inhibitory concentration (IC_50_) assessment for citalopram at 0.5 and 5 mM concentrations of acetylthiocholine and butyrylthiocholine as substrates of AChE and BChE, respectively. (C) The corresponding IC_50_ assessment for escitalopram against AChE and BChE. All data are shown as the percent of the enzymes’ activity in the presence of the solvent (vehicle control). Data are shown as mean ± SD. The IC_50_ analyses were done with GraphPad Prism 9.

A similar *in vitro* screening was also done for citalopram and its *S*-enantiomer, escitalopram, against both AChE and BChE (Figure 2A). The result indicated that both citalopram and escitalopram inhibited AChE by about 60%. The corresponding analysis on plasma BChE indicated that citalopram also inhibited the BChE activity by about 55%, while escitalopram showed a mild inhibition of BChE (∼10%, Figure 2A). The subsequent IC_50_ analyses confirmed that citalopram behaves as a dual inhibitor of human AChE and BChE (Figure 2B), while escitalopram is more selective toward AChE than BChE (Figure 2C).

**Figure 3:**
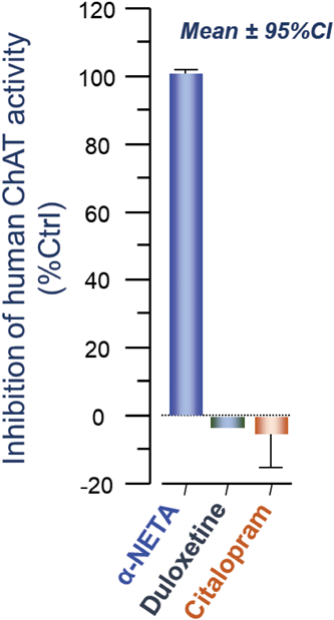
*In vitro* screening of citalopram and duloxetine against human recombinant choline acetyltransferase. The screening of duloxetine and citalopram against human choline acetyltransferase (ChAT) at a single concentration of 100 μM. None of the drugs show any activity against ChAT. We used α-NETA, a known inhibitor of ChAT, as the positive control. Data are shown as mean ± 95% confidence interval.

We also screened the drugs against human ChAT (Figure 3). This analysis indicated that neither duloxetine nor citalopram/escitalopram affected the activity of the human ChAT protein (Figure 3). Therefore, no further enzyme analyses were done for these drugs on ChAT.

### *Ex Vivo* Enzyme Inhibition Kinetic Analyses

**Figure 4:**
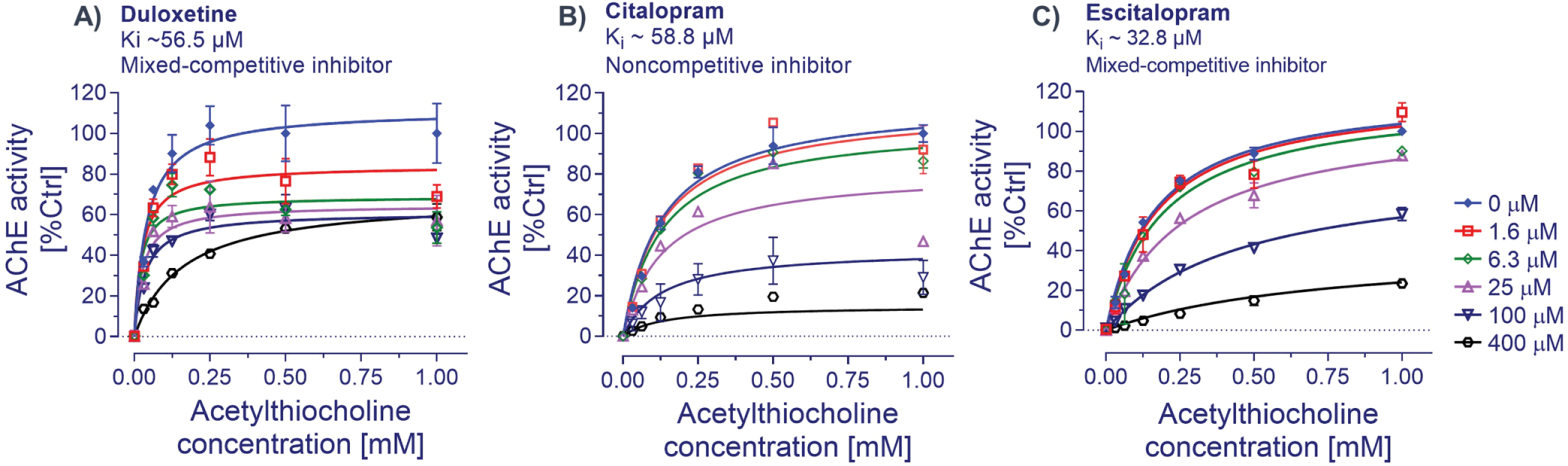
Enzyme inhibition kinetic analysis of duloxetine against human acetylcholinesterase. (A) Nonlinear regression analyses estimated an inhibition constant (*K*_i_) of 65.5 μM for duloxetine against human AChE. Duloxetine behaved as a mixed-competitive inhibitor of AChE. Similar analyses on (B) citalopram and (C) its *S*-enantiomer, escitalopram, estimated *K*_i_ values of 58.9 and 32.8 μM, respectively. The analyses in addition indicated that citalopram behaves like a noncompetitive inhibitor, while escitalopram is a mixed-competitive inhibitor like duloxetine. Thus, escitalopram appears twice as potent an AChE inhibitor as duloxetine and citalopram. Nonetheless, the *K*_i_ values suggest that all three drugs have moderate potency toward human AChE. Data are given as mean ± SD. The nonlinear regression analyses were done with GraphPad Prism 9.

**Figure 5:**
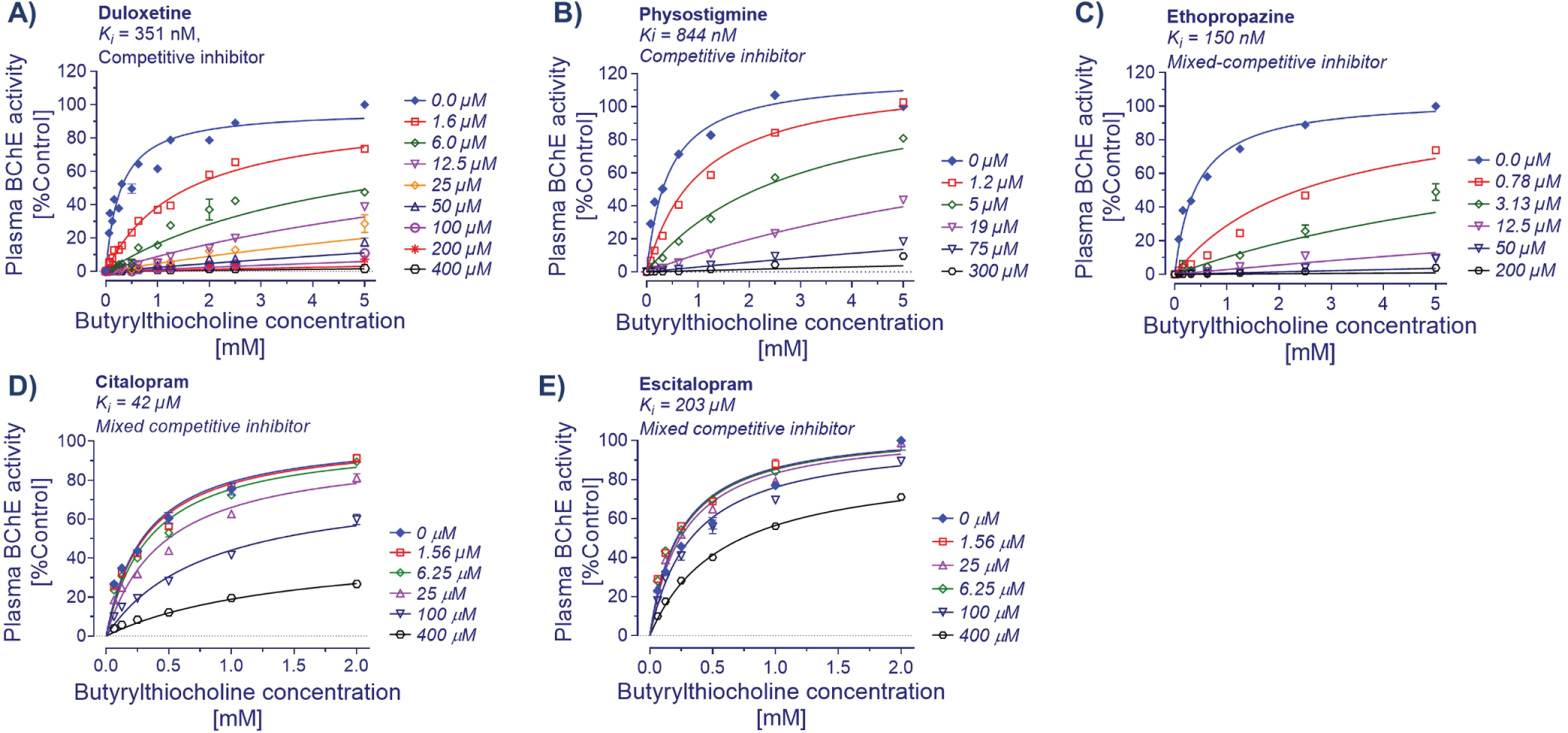
Enzyme inhibition kinetic analysis of duloxetine against human butyrylcholinesterase. (A) Duloxetine acts as a potent inhibitor of human BChE with a competitive mode of action. Nonlinear regression analyses estimated an inhibition constant (*K*_i_) of 351 nM for duloxetine against human BChE. (B) Similar nonlinear regression analyses indicate that physostigmine, a well-known nonselective cholinesterase inhibitor, has in comparison to duloxetine over 2-folds less affinity for human plasma BChE, as it can be deduced by a *K*_i_ value of 844 nM. (C) Ethopropazine, a well-known, highly potent, and selective BChE inhibitor, exhibits a *K*_i_ value of 150 nM in similar analyses in our laboratory. A comparison of the *K*_i_ value of ethopropazine with that of duloxetine indicates that duloxetine is indeed a potent inhibitor of human plasma BChE. (D, E) Nonlinear regression analyses indicated that in contrast to duloxetine, citalopram and its pure enantiomer, escitalopram, were much weaker BChE inhibitors, as is deduced by their estimated *K*_i_ of 42 and 203 μM against human BChE, respectively. The nonlinear regression analyses were done with GraphPad Prism 9. Data are given as mean ± SD.

Given that IC_50_ is a parameter that can greatly be affected by the concentration of the substrate and given that *in vivo* it is unlikely that the acetylcholine concentration reaches such a high concentration that was used here, we performed enzyme inhibition kinetics and assessed the inhibition constant (*K*_i_) for duloxetine, citalopram, and escitalopram against human AChE (Figure 4) and BChE (Figure 5). These analyses estimated a *K*_i_ of 65.5 μM (with a 95% CI of 27.9–226.8 μM) for duloxetine against human AChE (Figure 4A). The analyses further indicated that duloxetine behaved like a mixed-competitive inhibitor of AChE. The corresponding *K*_i_ of citalopram for AChE was 58.8 μM (with a 95% CI of 46.9–74.3 μM, Figure 4B), while escitalopram exhibited a *K*_i_ of 32.8 μM (with a 95% CI of 27.4–52.4 μM, Figure 4C). The analyses also indicated that escitalopram behaved (like duloxetine) as a mixed-competitive inhibitor, while citalopram acted like a noncompetitive one.

The enzyme inhibition kinetic analyses on BChE indicated that duloxetine is a potent competitive inhibitor of human BChE with a *K*_i_ of about 351 nM (with a 95% CI of 336–399 nM, Figure 5A). Comparatively, duloxetine was over 2-folds a stronger inhibitor of BChE than physostigmine, which exhibited a *K*_i_ of 844 nM (with a 95% CI of 709–979 nM, Figure 5B). For additional comparison, we also estimated the *K*_i_ for ethopropazine, a known highly selective and potent inhibitor of BChE. Ethopropazine behaved as a mixed-competitive BChE inhibitor with a *K*_i_ of 150 nM (with a 95% CI of 125–176 nM, Figure 5C).

The corresponding BChE inhibition kinetic analyses for citalopram and escitalopram estimated a *K*_i_ of 42.0 μM (with a 95% CI of 31.9–57.2 μM, Figure 5D) for citalopram and a *K*_i_ of 203.0 μM (with a 95% CI of 146.1–349.5 μM, Figure 5E) for escitalopram. Both compounds behaved like mixed-competitive inhibitors of BChE.

### Duloxetine Acts as a Competitive BChE Inhibitor

**Figure 6:**
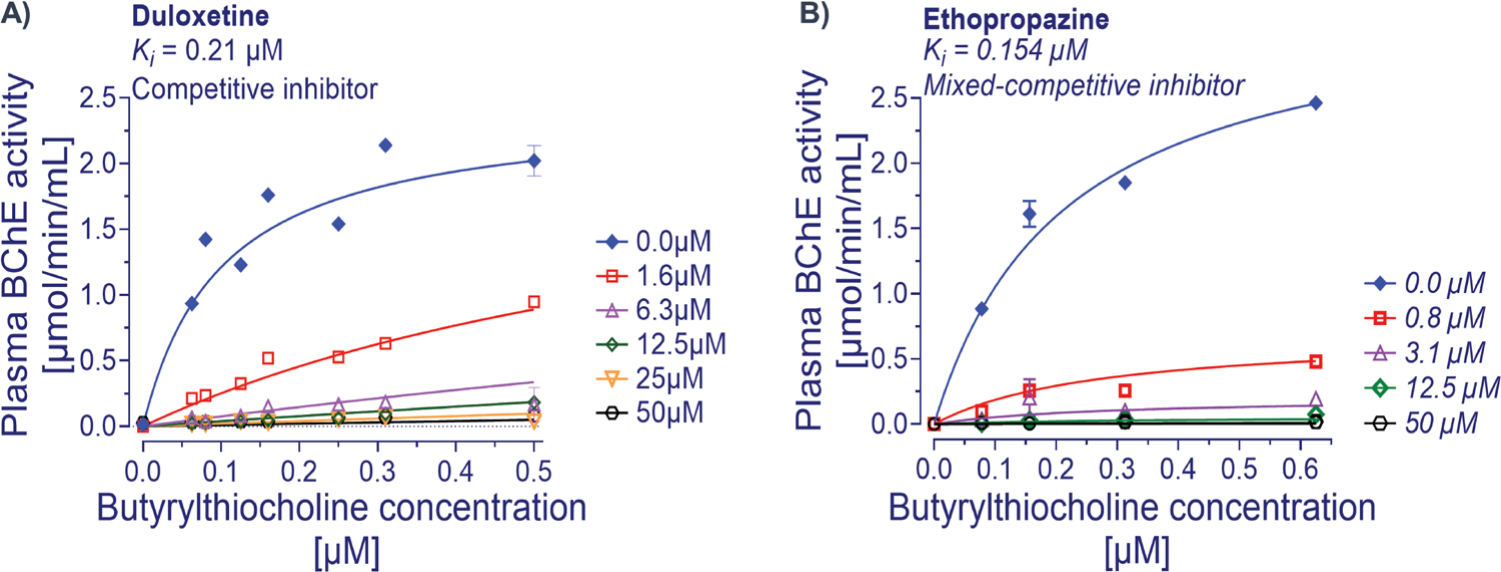
Enzyme inhibition kinetic analysis of duloxetine against human butyrylcholinesterase. (A) Duloxetine is most potent at low substrate concentrations (up to 0.5 mM) with a *K*_i_ of 0.21 μM compared to a high substrate concentration of up to 5 mM (see Figure 5A). This is most likely due to duloxetine being a competitive BChE inhibitor. (B) Similar analyses at low substrate concentrations indicate that the ethopropazine affinity is not affected (see Figure 5C). This is because it acts as a mixed-competitive inhibitor. Nonlinear regression analyses were done with GraphPad Prism 9. Data are given as mean ± SD.

Given that the enzyme inhibition kinetics of duloxetine indicated a competitive mode of action and that it is very unlikely that the *in vivo* concentration ranges of ACh reaches the high millimolar substrate ranges that were used in the initial assessment, we repeated the analyses in micromolar ranges of the substrate. The result indicated that duloxetine, as was expected, behaved like a competitive BChE inhibitor with the *K*_i_ value that was reduced by over 2-folds to 210 nM (compare Figure 6A with Figure 5A). In contrast, ethopropazine, being a mixed-competitive inhibitor, maintained its inhibition constant when assessed at the micromolar substrate range (∼155 nM, comparing Figure 6B with Figure 5C).

### Molecular Docking

Molecular docking has been proven to be a powerful tool in the field of computational drug discovery. Molecular docking analysis provides information about the interaction between a compound and the target protein (enzymes or receptors). For instance, it provides docking scores, in −log values, as an estimation of the binding affinity of the ligand to the target enzyme or receptor. A lower docking score (more negative) suggests a high binding affinity, thereby allowing to rank the compounds in a large chemical database. In the current study, we first performed a general structure-based virtual screening protocol based on the molecular docking analysis of all of the FDA-approved drug databases against human BChE, AChE, and ChAT. Duloxetine (−8.658 kcal/mol), citalopram (−8.669 kcal/mol), and escitalopram (−9.095 kcal/mol) were among the top hits as potential BChE ligands. These were then subjected to *in vitro* screening and *ex vivo* analyses.

**Figure 7:**
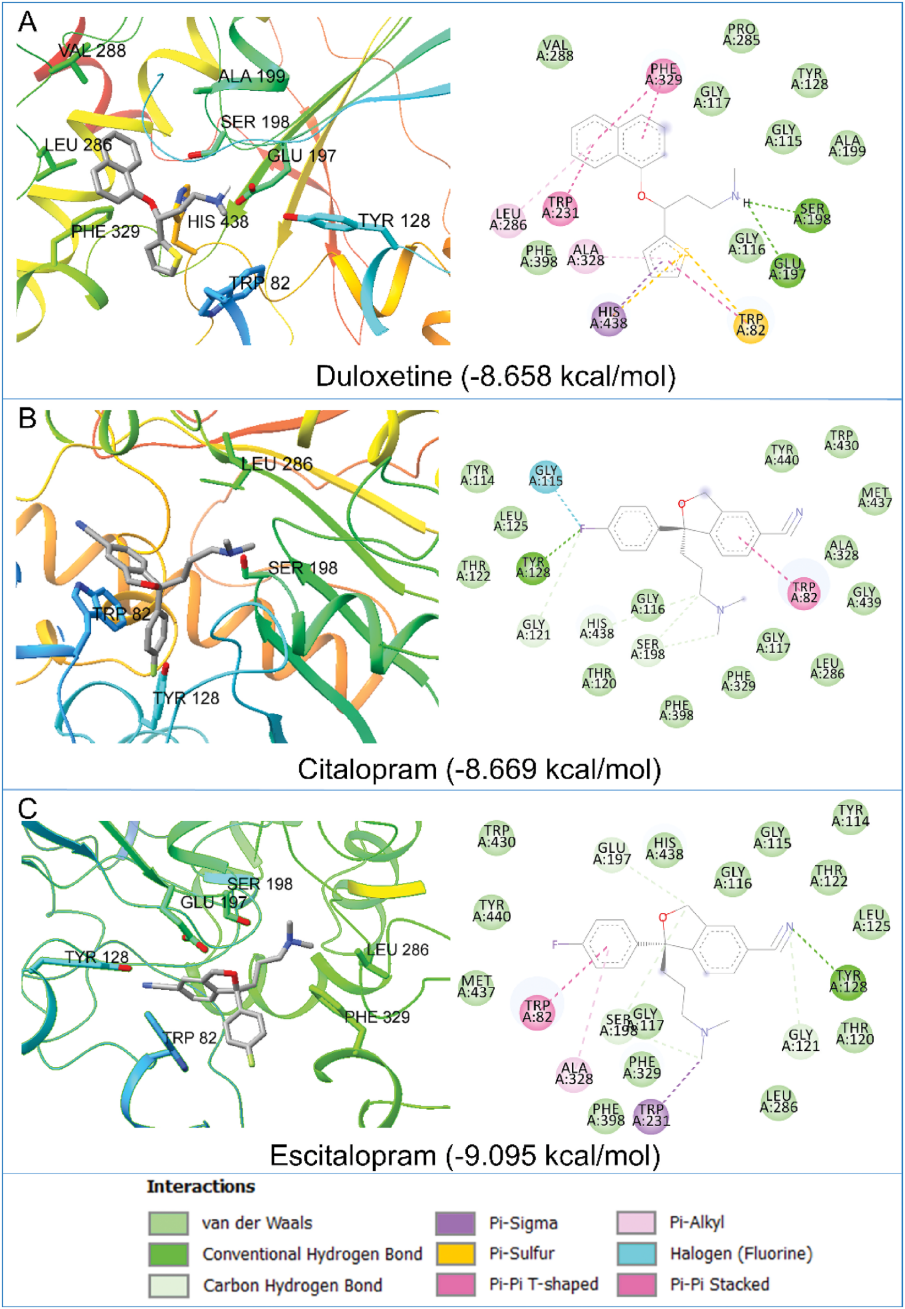
Molecular interaction of (A) duloxetine, (B) citalopram, and (C) escitalopram with human butyrylcholinesterase (BChE, PDB ID: 4BDS) obtained through molecular docking analysis. Three-dimensional (3D) and 2D interactions of these drugs with the binding site amino acid residues are shown. The dotted lines in the 3D diagram show the van der Waals interactions taking place between the protein and the ligand.

We next carried out molecular docking studies on duloxetine, citalopram, and escitalopram to understand various interactions at play between the compounds and the target proteins. The compounds were docked onto BChE as the target protein (PDB ID: 4BDS). The two-dimensional (2D) interaction diagrams along with the docking scores are shown in Figure 7. The active site of BChE consists of three different parts constituting several important amino acid residues. The catalytic anionic site (CAS) includes the amino acid residues Glu197, Ser198, Glu325, and His438. The peripheral anionic site (PAS) consists of Asp70, Trp82, Tyr332, and Tyr128 residues. The midgorge site (MIG) is located between the CAS and PAS regions and consists of residues Leu286, Val288, Ala199, and Phe329. MIG plays a crucial role in accommodating the acyl portion of the substrate.

These analyses indicated that duloxetine interacted with BChE (1) with a conventional hydrogen bond with the Glu197, Ser198, and His438 residues with a Pi–σ bond of the CAS site, (2) with the residue Trp82 in a Pi–sulfur bond, Tyr128 with van der Waals interaction of the PAS site, and (3) with Leu286 with the Pi–alkyl bond, Val288 and Ala199 with a van der Waals interaction and Phe329 with a Pi–Pi stacked interaction at the MIG site (Figure 7A). Likewise, citalopram interacted (1) with Ser198 as a carbon hydrogen bond at the CAS site, (2) with Trp82 by a Pi–Pi stacked interaction, and Tyr128 with a conventional hydrogen bond at the PAS site, and (3) with Leu286 by a van der Waals interaction in the MIG region (Figure 7B). The escitalopram interaction with BChE (Figure 7C) occurred (1) with Glu197 and Ser198 with a carbon hydrogen bond at the CAS site, (2) with Trp82 *via* a Pi–Pi stacked interaction, and Tyr128 by a conventional hydrogen bond at the PAS site, and finally (3) with Leu286 and Phe329 by van der Waals interactions at the MIG site. Noteworthily, all of the three compounds also showed a good interaction with the rest of the amino acid residues lying in the CAS, PAS, and MIG regions of the BChE protein.

Overall, the *in silico* docking analyses suggested that duloxetine, citalopram and escitalopram should have a comparable affinity to BChE, while the *ex vivo* enzyme inhibition kinetic analyses demonstrated that duloxetine had a 120- and 580-folds higher BChE inhibitory activity compared to citalopram and escitalopram, respectively. These discrepancies in the predicted binding affinities between the *in silico* docking and the enzyme inhibition kinetic analyses might be due to the limitations inherent in molecular docking, like the simplified representation of molecular interactions, the inability to fully replicate the complex molecular environment, or both. However, the 2D docking interactions suggested that duloxetine interacted with more amino acid residues in the CAS, PAS, and MIG sites of BChE, which might explain its superior inhibitory activity on the enzyme. In addition, we also performed docking analysis on AChE and ChAT, as presented in the Supporting Data. Overall, the *in silico* results were in line with the *in vitro* findings.

## Discussion

We showed that duloxetine possesses a potent secondary mode of action as a BChE inhibitor with a potency (inhibition constant) that lies in the range of its pharmacological plasma concentration in humans. In fact, duloxetine seems to be as potent a BChE inhibitor as ethopropazine, one of the most potent known inhibitors of this enzyme. In addition, we found that duloxetine was 4 times as potent as physostigmine with regard to inhibiting the activity of BChE.

Another study, using a different approach, identified several drugs, including duloxetine, that inhibited human BChE with over 70% at a concentration of 10 μM.^24^ This report estimated an IC_50_ of 1200 nM for duloxetine against human BChE, which greatly differs with the *K*_i_ of 210 nM, estimated for duloxetine by the *ex vivo* enzyme inhibition kinetic studies in the current report (Figure 6). This discrepancy is most likely due to the fact that IC_50_ is determined at a single concentration of the substrate and that IC_50_ estimation (in contrast to *K*_i_) may greatly vary depending on the substrate concentration. Unfortunately, Chrétien et al.’s paper neither clearly reports which substrate (butyrylthiocholine or acetylthiocholine) was used nor does it report the actual concentration of the substrate for the estimation of IC_50_. However, the *in vitro* BChE activity is most often measured at 1.0 or 5.0 mM using butyrylthiocholine as the substrate. Indeed, we found an IC_50_ of 9.7 μM for duloxetine at 5 mM substrate concentration (Figure 1B). Given the lack of reliability of IC_50_ estimates, we performed enzyme inhibition kinetic analysis, which, in addition to the much more reliable inhibition constant (*K*_i_), provides another important pharmacological parameter that IC_50_ analysis cannot. This is the determination of the mode of inhibition of the enzyme by the inhibitor, i.e., whether the inhibitor interacts with the enzyme through a competitive, noncompetitive, uncompetitive, or mixed-competitive mode of action. This information is crucial for judging whether a drug may be able to exert a meaningful *in vivo* effect on its target. We report here that duloxetine behaves as a fully competitive inhibitor of the human plasma BChE with a *K*_i_ of 210 nM that is in the normal *in vivo* concentration range of 100–400 nM duloxetine in human patients.^25,26^

As will be discussed, BChE together with AChE coregulates the extrasynaptic cholinergic signaling involved in controlling immune cells and astroglia cells.^4^ The *in vivo* extracellular acetylcholine concentration in blood and the CSF is less than 50 nM.^27,28^ At such *in vivo* concentrations, it is very unlikely that acetylcholine could displace duloxetine from their mutual binding site in the catalytic gorge of the enzyme. Thus, with a *K*_i_ of 210 nM in the 0–0.5 mM substrate range (Figure 6A) and an *in vivo* concentration of 100–400 nM,^25,26^ duloxetine is expected to have a full pharmacological activity as a BChE inhibitor. The normal concentration of acetylcholine in the synaptic cleft may however be much higher and in the 1–3 mM range.^29^ Nonetheless, duloxetine with a *K*_i_ of 350 nM in the high substrate range of 0–5 mM (Figure 5A) is still expected to exert a pharmacologically meaningful *in vivo* synaptic inhibition of BChE. Altogether, duloxetine at its normal dosages should be able to exert a cholinergic enhancing effect, especially in the elderly.

This finding has important clinical implications since accumulating reports indicate that BChE could be a legitimate target enzyme for improving cognitive function in patients with impaired cognition related to a deficit in the cholinergic system.^4,5^ For instance, BChE is fully capable of regulating the cholinergic signaling by hydrolyzing acetylcholine, i.e., in the same manner as AChE.^4,7^ In addition, genetic studies on BChE variants with a 30–60% reduced catalytic activity show that carriers of such variants exhibit a delay in developing cognitive impairment.^4,30^ It has been suggested that such genetic variants cause an intrinsic *in vivo* condition that resembles that of individuals being on treatment with a selective BChE inhibitor.^31^ Intriguingly, an 8-week double-blind placebo-controlled trial reports that duloxetine in addition to its effect on depression and some measures of pain also improved cognition in the elderly.^13^ Additionally, in a 12-week study in patients with major depressive disorders, duloxetine treatment resulted in significant cognitive improvements across several domains. Finally, a pharmacoepidemiologic study indicates that patients with depression who were treated with duloxetine had a significantly lower risk of developing dementia compared to those treated with citalopram.^32^

In another study, we have reported that variability in the BChE activity is closely related to the immune-regulatory role of ACh on astroglia function in the brain.^1^ Furthermore, there is evidence from our studies that the BChE activity is modified by the levels of amyloid-β (Aβ) peptides through the formation of a complex named BAβACs.^33^ BChE becomes hyperactivated upon the formation of this complex, leading to a shift in a tightly maintained extracellular ACh equilibrium^34,35^ that plays a crucial role in regulating the functional status of diverse cholinoceptive excitable and nonexcitable cells in circulation and in the brain, such as astroglia, which for instance dictate the inflammatory responses in the brain.^1,3,36^ We have hypothesized that BChE rather than AChE is involved in a proper activation and maintenance of a protective astroglia functional status in the brain.^1^ Overall, regulation of the BChE activity has been considered a prominent therapeutic target^5,37^ but so far little progress has been made in developing a selective BChE inhibitor. Here, we provided essential evidence indicating that duloxetine with an affinity level of ∼210 nM should be expected to affect the BChE activity within its expected *in vivo* concentration ranges of 100–400 nM following its conventional dosing regimen.^18,25,26^

Furthermore, advancing age is accompanied by a gradual deficit in cholinergic signaling that may predispose the elderly to various disturbances such as a reduced efficiency in response of the immune system and the processes involving the resolution of inflammatory cascades. There is also overwhelming evidence indicating that a deficit in the cholinergic system is not merely a consequence of the β-amyloidopathies and tauopathies seen in the major dementia disorders, like AD and Lewy body dementia, but one of the driving forces in these diseases.^22,38,39^

Similarly, depression is relatively common among elderly populations, with the manifestation of cognitive deficit.^12,13^ Depression is also a common comorbidity in MCI patients and a risk factor for the progression to dementia.^40^ Furthermore, depression is one of the most prevalent psychological symptoms in patients diagnosed with dementia, which is often underdiagnosed and undertreated.^41^ Other treatment indications for duloxetine are chronic skeletomuscular pain and stress-induced incontinence that is prevalent among the elderly. In all of these cases, duloxetine in addition to its primary antidepressive effect may offer beneficial clinical outcomes, such as improved cognition and prevention against dementia by augmenting the cholinergic function through its secondary mode of action as a BChE inhibitor. This possible cholinergic enhancing effect of duloxetine may also have a positive effect on the declining function of the immune system observed in elderly people.^42,43^ Indeed, the increased cholinesterase activity is associated with a decline in the cholinergic anti-inflammatory signaling^2^ and a low-grade systemic inflammation.^3^

The current treatment of patients with AD consists of three cholinesterase inhibitors, namely, donepezil, galantamine, and rivastigmine. Among these, both donepezil and galantamine are highly selective against AChE. Given that at therapeutic concentrations, donepezil and galantamine are completely devoid of any inhibitory effect on BChE,^44−47^ and since BChE can compensate for the reduced AChE activity,^4^ concomitant treatment with duloxetine may offer an additive or synergistic cholinergic enhancing effect by negating such a compensation effect of BChE.^48^ Nonetheless, duloxetine may exert a pharmacodynamic additive effect with rivastigmine, a pseudoirreversible (slowly reversible) inhibitor of both AChE and BChE.^49^ Therefore, care must be taken in patients treated with this drug regarding concomitant duloxetine treatment to avoid possible side effects due to hyperexcitation of the cholinergic system. Similarly, elderly subjects with a K- or J-variant of BChE may require a careful introduction to duloxetine treatment, given that K and J mutations in the BCHE gene are often present together and result in a 30–60% intrinsic reduction in the BChE activity.^6^ There are also other phenomena that may arise due to a molecular interaction between high apolipoprotein E (which is seen in patients with the ε4 allele of APOE4) that results in a reduced BChE activity,^50^ as well as a possible synergistic effect of BCHE-K and APOE4 on the development of AD.^51^

We also report here that citalopram and its enantiomer, escitalopram, were moderate inhibitors of AChE and BChE. As an AChE inhibitor, escitalopram was twice as potent as citalopram (*K*_i_^Cit^/ *K*_i_^Escit^ = 59/33 = 1.8). However, as a BChE inhibitor, citalopram was about 5 times more potent than escitalopram (*K*_i_^Escit^/*K*_i_^Cit^ = 203/42 = 4.8). The concentration of citalopram varies depending on the dosage, but ranges between 28 and 279 ng/mL in the plasma and between 13 and 96 ng/mL in the CSF.^19^ The concentrations can be slightly higher in elderly populations than in young people.^20^ The mean plasma concentration of escitalopram is fairly similar to that of citalopram and varies depending on the dosage (63 and 198 ng/mL, following repeated doses of 10 and 30 mg/day, respectively^21^). Using a molecular weight of 325.39, the molar concentrations of citalopram and escitalopram are at most about 0.86 μM. This concentration is much lower than the *K*_i_ values for citalopram and escitalopram as AChE or BChE inhibitors. Therefore, it is unlikely that citalopram or escitalopram can induce any significant *in vivo* inhibition of these two enzymes in the peripheral or central nervous system.

In conclusion, duloxetine was found to be a potent BChE inhibitor with a highly probable *in vivo* effect, given that BChE can coregulate the cholinergic signaling by acting on acetylcholine,^4^ particularly in elderly populations or in cognitively impaired older adults in whom BChE levels may have been elevated.^4^ Duloxetine as a novel BChE inhibitor should be therefore considered the choice of treatment in older adults with both depressive and dementia symptoms.

## Materials and Methods

This work is in continuation of our previously published research.^22^ The *in silico* analysis was essentially done as described before.^22,23^ Briefly, we initially screened a database of FDA-approved drugs primarily against BChE, AChE, and ChAT with the help of the SYBYL-X 2.1.1 (SYBYL-X 2.1.1, Tripos International, 1699 South Hanley Rd., St. Louis, MO 63144) molecular modeling suite installed on the Linux-based Dell Precision T7610 workstation [Intel Xeon E5-2643 CPU@3.3 GHz; 16 GB RAM, 2 TB hard disk].^22^ Nonetheless, some of the hits showed a considerable docking score against BChE as compared to AChE and ChAT. The compounds that were commercially available were purchased and subjected to a detailed *in vitro* inhibition assay against all three enzymes, namely, ChAT, AChE, and BChE. This resulted in the identification of some potent BChE inhibitors with varying degrees of inhibitory activity also toward AChE and ChAT.^22^ In order to deduce the binding interaction to the active site and the involved amino acid residues, we also carried out targeted *in silico* analyses of duloxetine, citalopram, and escitalopram against BChE, AChE, and ChAT.

### Molecular Docking

Molecular docking is a fast and inexpensive tool that has been widely used in the field of drug design and discovery for predicting the binding modes and estimating the binding affinity.^52^ It involves computationally predicting the binding orientation, i.e., pose generation of a small-molecule ligand within a protein’s binding site, followed by scoring to evaluate the strength and feasibility of the protein–ligand interaction based on various physical and chemical criteria. These obtained scores (kcal/mol in the case of AutoDock) provide crucial information about the change in the binding free energy, where usually a lower docking score denotes more favorable binding.^53^ The molecular docking studies can predict the binding pose of the compounds with reasonable accuracy in comparison to its experimental binding pose, thus making it an ideal tool to understand the crucial binding interactions taking place during complex formation.^54^ Here, we have utilized Autodock Vina software^55^ for carrying out the molecular docking studies in order to understand the possible protein–ligand interactions. The 3D X-ray crystallographic protein structures were obtained from the RCSB Protein Data Bank (PDB)^56^ for human BChE (PDB ID: 4BDS), AChE (PDB ID: 4EY7), and ChAT (PDB ID: 2FY3). The protein structures were first prepared by clearing residual water, ions, and other ligand molecules, followed by the addition of polar-only hydrogens, assigning AD4-type atoms and Kollman charges. Then, it was converted to a suitable (pdbqt) format for docking by utilizing the edit features present in AutoDock Tools 1.5.6.^57^ The chemical structures of duloxetine, citalopram, and escitalopram were converted as well to a suitable (pdbqt) format for docking.

The grid box was prepared with the cocrystallized ligand as the center coordinate with a box size of 26 × 26 × 26 with a grid point spacing of 1 Å and the exhaustiveness was set to 8 as default for all of the three systems. The compounds were docked into the binding site and the binding energy (kcal/mol) was recorded for the ligands. The obtained docking pose was visualized for their conformation and interaction using a PyMOL viewer.^58^

### Measurement of Inhibition of AChE and BChE Activity

The screening of the *in silico* hits toward the BChE and AChE enzymatic activity was done by an adapted high-throughput assay version of Ellman′s colorimetric assay.^59,60^ The reagents, butyrylthiocholine iodide (BTC), acetylthiocholine iodide (ATC), and 5,5′-dithiobis(2-nitrobenzoic acid) (DTNB), were purchased from Sigma-Aldrich (St. Louis, MO). The modified assay details are as previously described.^6,44^ The main modification concerned the high-throughput adaptation of the assay for use in 384-well plates. Briefly, 25 μL/well of a 1:450 diluted solution of pooled human plasma and 1:768 diluted (3.5 ng/mL final concentration) purified recombinant human AChE protein (Sigma, Cat no. C1682) was used for measurement of the BChE and AChE activity, respectively. These enzymes’ concentrations were used since they are representative of the activity of these enzymes in the human cerebrospinal fluid (CSF).

For the *in vitro* screening, the order of steps was as follows: (1) 25 μL of the working solution of duloxetine, citalopram, or escitalopram was added in quadruplicates in the wells of a 384-well plate. To the control wells, the vehicle solution was added to get the reference enzyme activity (100%). (2) Then, 25 μL of a working solution of BChE or AChE was added to all wells. The plate was then incubated for 10–30 min at room temperature. (3) The reaction was then started by adding 25 μL of a cocktail mix prepared in Na/K phosphate buffer to all wells, containing DTNB (final concentration 0.4 mM) and BTC (final concentration 1 mM) or ATC (final concentration 0.5 mM). The changes in the absorbance were monitored at 412 nm wavelength for 15–20 min at 1 min intervals, using a microplate spectrophotometer reader (Infinite M1000, Tecan). The rate of the enzyme activity was determined from the slope of the linear part of the kinetic reaction curves. In all steps, the working solution concentrations were 3 times of the final concentrations of each component in the final volume of 75 μL in each well. The final screening concentration of the compounds was 100 μM. The stock concentration of the compounds was prepared in 100% dimethyl sulfoxide (DMSO) at 10 mM or more, allowing the final DMSO concentration in the wells to be less than 4%. The final concentration of DMSO was used as the vehicle control.

### *Ex Vivo* Kinetic Studies for the Estimation of IC_50_, *K*_i_, and the Mode of Action of the Drugs

For kinetic studies, a similar protocol as the screening assay was followed; a dilution series of five different concentrations ranging from μM to nM were prepared for duloxetine, citalopram, and escitalopram.

For BChE, the final BTC concentrations in the wells ranged from 5 mM to 156 nM, which were prepared as a 2-fold serial dilution. For AChE, the final ATC concentrations in the wells ranged from 0.5 mM to 31.3 nM, prepared as a 2-fold serial dilution series.

In all enzyme inhibition kinetic assays, the concentrations of AChE and BChE were chosen so that the enzyme activities were representative of the activity of these enzymes in the human CSF, which is about 20 and 10 nmol/min/mL CSF for AChE and BChE, respectively.^49^

For the enzyme inhibition kinetic analyses, the order of steps was as follows: (1) 25 μL/well of the compound or the vehicle control was added in quadruplicates, with respect to each compound concentration, in a 384-well plate. (2) Then, 25 μL of each substrate concentration was added to the wells in quadruples. (3) The reaction was then started by adding 25 μL of a master mix to all wells, containing the enzyme working solution and DTNB. The final concentration of the enzyme and DTNB as well as the buffer composition was the same as mentioned above for the screening assay. Then, the plate was immediately placed in the microplate spectrophotometer reader (Infinite M1000, Tecan) and the changes in the absorbance were continuously monitored at 2 min intervals for 15–20 min at 412 nm wavelength. The rate of the enzyme activity was extracted as the slope of the initial linear part of the kinetic reaction curves.

The data were then transferred and processed using GraphPad Prism 9 analysis software.^15^ The half-maximal inhibitory concentration (IC_50_) values were calculated by plotting the percentage enzyme activity versus the log concentrations of the compound. The inhibitory constant (*K*_i_) values were determined from the enzyme inhibition kinetic analyses using the nonlinear regression function in the software. A statistical comparison of the ligand’s mode of activity was also done using GraphPad software between the competitive equation versus the mixed-competitive, noncompetitive, and uncompetitive equations.^61^ A competitive mode of action means that the inhibitor reversibly binds to the substrate′s binding site. Thus, the inhibition of the enzyme is modifiable by a high concentration of the substrate *via* displacement. A noncompetitive mode of activity differs since in this case, the inhibitor reversibly binds to both the enzyme–substrate complex and the enzyme itself, meaning that the ligand and the substrate do not compete for the same binding site. In the case of an uncompetitive mode of activity, the inhibitor has an affinity to the enzyme–substrate complex, but not the free enzyme. When the behavior of a ligand is not fully uniform, and the mixed-competitive fitting equation is statistically the best fitting option, the ligand is called a mixed-competitive one. This is determined through a general equation that includes the other three modes of actions. Through this equation, the software provides an additional parameter, called α, which determines the mechanism of action of the inhibitor. For instance, when α is equal to one, the inhibitor′s affinity for the free enzyme and the enzyme–substrate complex is equal, i.e., the inhibitor behaves mainly noncompetitively. When α is greater than 1, the inhibitor has its peak affinity for the free enzyme. When α is very large, the inhibitor behaves mainly as a competitive ligand. A very small α (but not <0) is indicative of an uncompetitive mode of action.^61^ Depending on the contexts, these different modes of actions may have different *in vivo* pharmacological and pharmacodynamic outcomes, as is noted in the Discussion section.

## Supporting Information Available

Additional experimental details, materials, and methods, including supplementary figures of molecular docking of the compounds on AChE and ChAT (PDF)
