# Supplementary material for: Repurposing Duloxetine as a Potent Butyrylcholinesterase Inhibitor: Potential Cholinergic Enhancing Benefits for Elderly Individuals with Depression and Cognitive Impairment
Source: ACS Omega. 2024 Aug 21;9(35):37299–309. doi: 10.1021/acsomega.4c05089 (PMC11375813; doi:10.1021/acsomega.4c05089)
Supplement: Supplementary file 1 — ao4c05089_si_001.pdf [file ao4c05089_si_001.pdf]

Repurposing duloxetine as a potent butyrylcholinesterase inhibitor — *Potential cholinergic enhancing benefits for elderly individuals with depression and cognitive impairment.*

Taher Darreh-Shori[1*], Anurag TK Baidya[2], Medea Brouwer[1], Amit Kumar[1] and Rajnish Kumar[2].

[1]Division of Clinical Geriatrics, Centre for Alzheimer Research, Department of Neurobiology, Care Sciences and Society, Karolinska Institutet, NEO,7th Floor, 141 52 Stockholm, Sweden

[2]Department of Pharmaceutical Engineering & Technology, Indian Institute of Technology (B.H.U.), Varanasi-221005 (U.P.) India

Correspondence: Taher Darreh-Shori, e-mail: taher.darreh-shori@ki.se

Molecular docking analysis against AChE and ChAT

Since AChE and ChAT are enzymes related to BChE, we performed docking analyses of these compounds on both enzymes. Like BChE, the active site in the human AChE contains three regions. CAS region is surrounded by Trp86, Tyr119, Tyr124, Tyr133, Glu202, Ser203, Trp439, His447, Tyr449 together with a series of glycine residues and PAS is in contact with Trp286, Tyr341, Val365, Tyr72, Tr75 and Leu289 residues. The mid-gorge site of human AChE is characterized by the Asp74, Leu76, Phe297, Phe338, Phe295 and Arg296. Duloxetine showed a docking score of -9.649 kcal/mol for AChE (**Fig. S1A**). Duloxetine interacted 1) at the CAS site with Trp86 via a van der Waals interaction and Tyr124 with a conventional hydrogen bond, 2) at the PAS site with Trp286 with a Pi-Pi Stacked interaction, Tyr72, and Tyr341 with a van der Waals interaction, and 3) at the MIG site with Asp74, Phe297, Phe295, Phe338 and Arg296 with a van der Waals interaction (**Fig. S1A**). Toward AChE, citalopram exhibited a docking score of -10.288 kcal/mol and showed interaction 1) at the CAS site with Trp86, Glu202 and His447 in a conventional hydrogen bond interaction, and with Tyr124 and Ser203 with a carbon hydrogen bond. 2) At the PAS site, citalopram interacted with Tyr341 in a carbon hydrogen bond, Tyr72 with a conventional hydrogen bond, along with the amino acids Asp74 in a carbon hydrogen bond, Phe338 and Phe295 with a conventional hydrogen bond at the MIG site (**Fig. S1B**). The docking score for escitalopram toward AChE was -10.178 (**Fig. S1C**). At the CAS site, escitalopram interacted with Trp86 in a Pi-Pi Stacked interaction, Tyr124 in a carbon hydrogen bond, and His447 by van der Waals force. At the PAS site, the interaction with Trp286 was via van der Waals force, with Tyr341 by a Pi-Pi Stacked bond and with Asp74 through a halogen fluorine bond. At the MIG site, escitalopram interacted with Phe297, Phe338 and Arg296 via van der Waals forces, and with Phe295 by a conventional hydrogen bond (**Fig. S1C**).

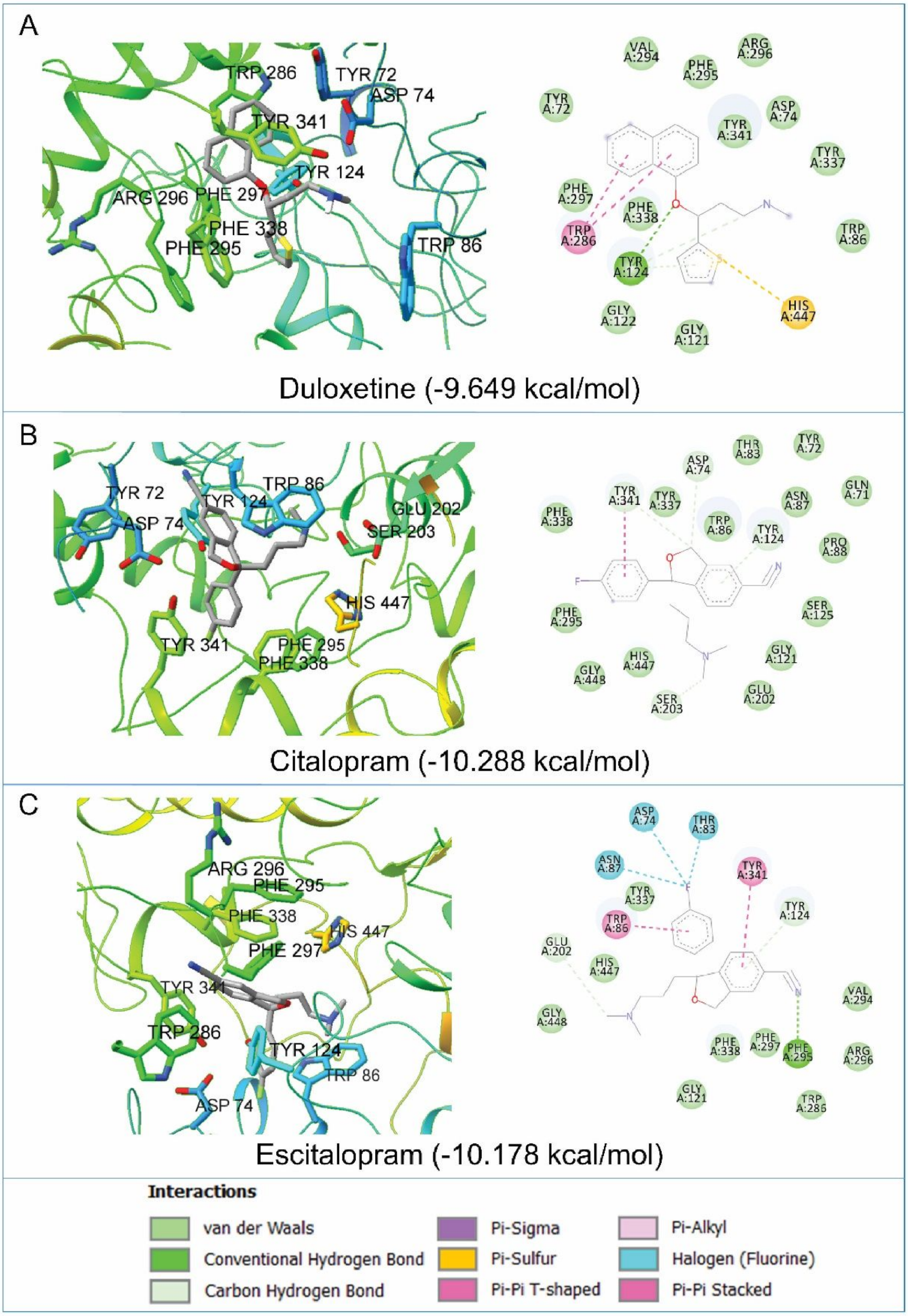

Figure S1. Molecular interaction of Duloxetine (A), Citalopram (B) and Escitalopram (C) with human acetylcholinesterase (AChE, PDB ID: 4EY7) obtained through molecular docking analysis. 3D and 2D interactions of these drugs with the binding site amino acid residues is shown. The dotted lines in the 3D diagram shows the van der Waals interactions taking place between the protein and ligand.

Here, the *in-silico* analyses suggested that duloxetine should be a weaker AChE inhibitor compared to citalopram or escitalopram which is in line with the enzyme inhibition kinetic analyses. In addition, the docking scores of escitalopram for AChE vs BChE were also in line with the *in vitro* enzyme-inhibition kinetic analyses indicating that escitalopram had much higher affinity toward AChE than BChE.

Finally, the compounds were also subjected to docking protocol against ChAT as it is the key cholinergic enzyme in the biosynthesis of acetylcholine. ChAT contains two binding cavities for its substrate, namely a choline/ACh binding site and an acetyl-Coenzyme A/ Coenzyme A binding site, and in between these two sites lies the catalytic amino acid residue His324, which is the most crucial amino acid residue for the catalytic function of the enzyme. However, the residues Tyr552, Asp390, Ser221, Arg403, Trp325 and Gly280 also play an important role in overall function of the enzyme.

The 2D representation of the docking interactions of duloxetine, citalopram and escitalopram are shown in **Figure S2**. Duloxetine exhibited a docking score of -7.304 kcal/mol (**Fig. S2A**). This drug did not interact with the key catalytic amino acid residue His324 or any other important residues of the ChAT binding tunnel, rather it seemed to be bound on the outer part of the choline binding site. On the other hand, citalopram with a docking score of-6.950 kcal/mol (**Fig. S2B**) interacted with the catalytic His324 amino acid residue through a Pi-Pi Stacked interaction with its 4-Fluro benzene ring (**Fig. S2B**) as it was binding at the choline binding site. However, like duloxetine, citalopram did not show any interaction with the other important residues of the ChAT catalytic tunnel. The interaction with His324 was absent in the docking results for the enantiomer, escitalopram, which exhibited a docking score of -6.476 kcal/mol for ChAT (**Fig. S2C**). Like citalopram, escitalopram did not interact with the other important amino acid residues of the ChAT binding cavity.

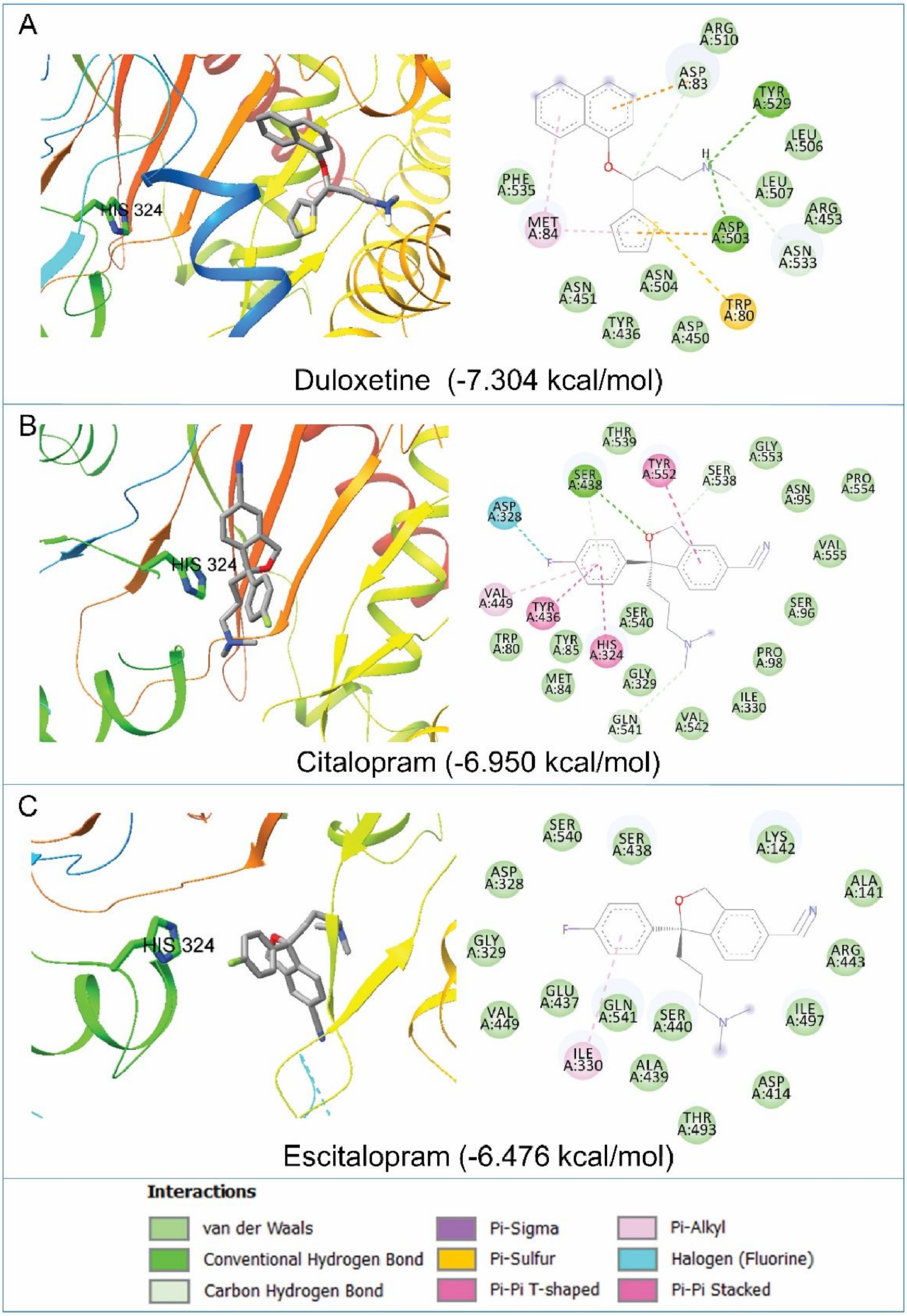

Figure S2. Molecular interaction of Duloxetine (A), Citalopram (B) and Escitalopram (C) with human choline acetyltransferase (hChAT, PDB ID: 2FY3) obtained through molecular docking analysis. 3D and 2D interactions of these drugs with the binding site amino acid residues is shown. The dotted lines in the 3D diagram shows the van der Waals interactions taking place between the protein and ligand.

In addition, escitalopram seemed to be bound at the acetyl-Coenzyme A binding site. Overall, the lower docking scores for ChAT relative to that for BChE and AChE and a lack of major interactions with the crucial amino acids on ChAT enzyme were well in accord with the *in vitro* data showing no significant inhibitory potencies for duloxetine, citalopram, and escitalopram (as depicted in **Fig. 3**).
